# Shift Work and Endocrine Disorders

**DOI:** 10.1155/2015/826249

**Published:** 2015-03-29

**Authors:** M. A. Ulhôa, E. C. Marqueze, L. G. A. Burgos, C. R. C. Moreno

**Affiliations:** ^1^Department of Medicine, UNEC, Nossa Senhora das Graças, Unity II, 35300-345 Caratinga, MG, Brazil; ^2^IMES, Rua João Patrício Araújo, No. 179 Veneza I, Ipatinga, MG, Brazil; ^3^Department of Epidemiology, Graduate Program of Public Health, Catholic University of Santos, Avenida Conselheiro Nébias 300, Vila Matias, 11015-002 Santos, SP, Brazil; ^4^Department of Environmental Health, School of Public Health, University of São Paulo, Avenida Dr. Arnaldo 715, Cerqueira César, 01246-904 São Paulo, SP, Brazil; ^5^Stress Research Institute, Stockholm University, 106 91 Stockholm, Sweden

## Abstract

The objective of this review was to investigate the impact of shift and night work on metabolic processes and the role of alterations in the sleep-wake cycle and feeding times and environmental changes in the occurrence of metabolic disorders. The literature review was performed by searching three electronic databases for relevant studies published in the last 10 years. The methodological quality of each study was assessed, and best-evidence synthesis was applied to draw conclusions. The literature has shown changes in concentrations of melatonin, cortisol, ghrelin, and leptin among shift workers. Melatonin has been implicated for its role in the synthesis and action of insulin. The action of this hormone also regulates the expression of transporter glucose type 4 or triggers phosphorylation of the insulin receptor. Therefore, a reduction in melatonin can be associated with an increase in insulin resistance and a propensity for the development of diabetes. Moreover, shift work can negatively affect sleep and contribute to sedentarism, unhealthy eating habits, and stress. Recent studies on metabolic processes have increasingly revealed their complexity. Physiological changes induced in workers who invert their activity-rest cycle to fulfill work hours include disruptions in metabolic processes.

## 1. Introduction

The production of goods and provision of services 24 hours a day, seven days a week, have risen dramatically over the last decade. This phenomenon has been largely attributed to rapid technological development, demographic characteristics, and globalization [1]. An estimated 20% of the economically active population in North America and Europe are engaged in some type of shifts involving night work [2]. There is considerable research interest among the scientific community regarding the mechanisms involved in health problems such as metabolic processes in this population.

Shift workers, more specifically night workers, tend to have unhealthy life habits, such as smoking, poor diet, and sedentarism [3].

The direct and indirect interrelationship of social problems and aspects related to misalignment of biological rhythms experienced by shift workers predisposes them to a number of deleterious health effects. Studies show that overweight and obesity are more prevalent in shift and night workers than day workers [4–6]. In addition, shift and night work have been associated with increased risk of developing other metabolic disorders, such as insulin resistance [7–10], diabetes [11–13], dyslipidemias [14–18], and metabolic syndrome [19, 20]. Another disruption reported in the literature is alteration in food intake [11, 21].

In general, the causes underlying shift and night work-related health problems are partially elucidated but warrant further investigation. Thus, the objective of this review was to investigate the impact of shift and night work on metabolic processes and the role of alterations in the sleep-wake cycle and environmental changes in the occurrence of metabolic disorders.

*Shift Work as a Disruptor of Circadian Control of the Endocrine System*. The temporal dimension was incorporated into living organisms during evolution as a means of adapting to environmental changes. Therefore, the expression of a given function of the organism can be fundamental for its survival by anticipating changes set to take place in the external environment. It is therefore clear that living beings oscillate endogenously according to the time of day and seasons of the year. These biological oscillations found in living beings which repeat regularly are referred to as biological rhythms, circadian rhythms, which occur within a period of approximately 24 h (±4 h) [1, 21–23].

The expression of biological rhythms takes place via interactions between central and peripheral endogenous molecular mechanisms, which enable the organism to adapt to changes in the external environment [24]. Circadian rhythms are aligned with periodic environmental events such as the light-dark cycle. In addition, circadian rhythms are interlinked; that is, there is a coupling of endogenous rhythms. These synchronized internal mechanisms ensure that all the physiological and behavioral rhythms occur in a coordinated fashion during the 24-hour cycle. This implies that the health of individuals whose timing between biological rhythms and environmental cycles is not kept in balance can be affected [25]. Desynchronization is defined as a change in the relationship of phases between two or more rhythms, a situation that can have a number of deleterious effects on health [26].

In mammals, the main circadian clock has been identified bilaterally in the suprachiasmatic nuclei (SCN) located in the hypothalamus. The main synchronizing agent of the central biological clocks in the control of circadian rhythms is the alternation between the light and dark cycle. Luminosity information is conveyed to the Central Nervous System (CNS) via the retinohypothalamic tract [8], where melanopsin plays the role of key photoreceptor in this process.

Driven by the light-dark cycle, the CNS orchestrates the circadian rhythms of many behaviors, tissues, hormones, and genes, as well as other physiological processes. In recent decades, studies have shown that molecular mechanisms occur in cells of peripheral tissues autonomously and that their circadian rhythms persist* in vitro* [27–29]. The synchronizers of peripheral clocks (located in organs, tissues, and cells) are neurohumoral factors such as glucocorticoids and melatonin, as well as social rhythms such as feeding and work times.

Control of expression of circadian rhythms also involves regulation at a cellular level through clock genes [1, 24]. High specificity of the clock genes in tissues also ensures perfect functioning of these complex intrinsic mechanisms, fundamental to trigger the physiological and behavioral responses [30].

Biological rhythms modulate practically all physiological processes of mammals where circadian control over the endocrine system has been shown to be of vital importance. However, alterations in alignment of environmental cycles with rhythms of the organism may induce alterations in the complex mechanism of hormonal and metabolic timing.

While some animals tend to be active during the night (e.g., rats), others such as humans are active predominantly during the day. However, humans are the only creature to voluntarily change their period of activity to nonhabitual times, forcing misalignment between activity phases and biological rhythms. As a consequence of this shift in activity times, changes take place in sleep times and feeding habits which together influence circadian control of the endocrine system and can have serious repercussions such as metabolic pathologies [31–33].

Among permanent night workers, less than 3% exhibited complete circadian adjustment [34], revealing that the majority of these workers experience misalignment of biological rhythms and its deleterious effects. In the ensuing text, some of the main metabolic disorders associated with shift and night work will be outlined.

## 2. Methods

The literature review was performed by searching the electronic database PubMed and for relevant studies published between January 2003 and March 2014 (lasting 10 years) and written in English. The methodological quality of each study was assessed, and best-evidence synthesis was applied to draw conclusions about the evidence for the effectiveness of each outcome.

The search terms were shift work, cortisol, insulin resistance, diabetes, obesity, and metabolic disorders (Table 1). Mostly included studies were with humans; however, others that had shown relevant conclusions regarding metabolic processes were included as well. Reviews were excluded from our results. Nevertheless, some reviews were used to support theories of the mechanisms involved in the misalignment's physiological changes in the discussion. To achieve the objective of the present paper, we mainly choose articles that have presented a rationale regarding metabolic disorders and endocrine system.

## 3. Results

### 3.1. Cortisol and Shift Work

One approach for determining the endogenous circadian rhythm is by measuring cortisol levels. Cortisol is a hormone produced by the adrenal gland whose functions encompass anti-inflammatory, metabolic (gluconeogenesis), and immunosuppressive responses [35–37]. Cortisol secretion by the adrenal gland is the result of a hormonal cascade commencing in the hypothalamus, more specifically in the paraventricular nucleus. This structure is situated in the CNS and also controls the secretion of the hormones of another gland located immediately below: the anterior hypophysis or pituitary. The hypothalamus secretes release factors into the blood stream stimulating the pituitary gland to secrete its hormones. Subsequently, after hypothalamic stimuli, the pituitary releases the adrenocorticotropic hormone (ACTH), whose function is to stimulate the adrenal gland to secrete cortisol. This is known as the hypothalamus-pituitary-adrenal (HPA) axis. Besides controlling release of cortisol, the hypothalamus also participates in controlling emotions, sleep, hunger, thirst, osmolarity, libido, and thermoregulation.

Stress stimulates the HPA axis [35, 38], which has a circadian rhythm that, under normal conditions, leads cortisol production to peak during early morning. The concentration of cortisol declines throughout the day, reaching its lowest level at night [35–37]. During the course of this cycle, which is well defined in the literature, there is also a marked cortisol peak between 20 and 30 minutes after morning awakening. This phenomenon is denoted by the cortisol awakening response (CAR) and appears to be a characteristic of the HPA axis, related to the circadian rhythmicity of cortisol [36].

There is a general consensus among studies on circadian rhythms that endocrine changes, such as those observed in shift workers, include alterations in cortisol concentration and its awakening response (CAR) [39, 40].

Sleep is a factor impacting cortisol concentrations. A laboratory-based study showed that subjects whose usual 12-hour sleep pattern was inverted from night to day experienced misalignment and inversion of their circadian rhythm. The authors implicated this effect as the cause for the glucose increase and cardiometabolic consequences that occur acutely in jetlag and chronically in shift work [37]. These circadian rhythms changes, including alterations in cortisol expression, have been associated with the development of cardiovascular diseases and cancer [41].

A pertinent question raised by the academic community concerns the type of shift that would be less harmful to workers' health. Both night and rotating shifts change cortisol level as compared to a daytime work schedule. Nevertheless, night shifts were found to more strongly associate with stressors at the work place [42]. Ulhôa et al. [40] also found a correlation between cortisol and stressors in irregular-shift work (performed as and when required, i.e., at any time of day or night). In the same study, a reduced cortisol awakening response in day workers during rest days compared to work days was found. However, this was not observed in irregular-shift workers, whose cortisol levels remained high during days off, evidencing a prolonged response to stress. A prolonged shift can also be a stressor [43].

In general, individuals who work very early mornings (morning shift) have partial sleep deprivation, obesity, and low morning cortisol concentration compared to those who work the afternoon shift [44]. Also, the study by Williams et al. [45] showed that waking up very early promotes an increase in the cortisol awakening response.

Kudielka et al. [46] compared the transition from night to rotating shifts in a field study. Cortisol concentrations were low during night shifts and remained unchanged when subjects changed over to rotating shifts. However, when the workers of the day time shift switched to the permanent night shift, cortisol levels were initially reduced and tended to normalize after an adaptation period.

The results in the literature are conflicting regarding neuroendocrine measures and type of rotating shift. A delay in acrophase and a lower amplitude were observed in the temperature rhythms of workers on counterclockwise rotating shifts compared to data for clockwise rotating shifts [47]. Another study conducted in workers on a counterclockwise rotating shift showed high cortisol levels compared to those found in personnel working permanent morning and night shifts [48]. In addition, the same study showed worse sleep quality in participants working rotating shifts. By contrast, the study of de Valck et al. [49] showed similar cortisol levels for counterclockwise and clockwise rotating shifts.

Exposure to stress is known to disrupt cortisol response and this should be taken into account when conducting studies on biological rhythms [50, 51]. Some authors hold that working shifts, per se, are a stressor [40]. Circadian rhythm misalignment, including alteration in cortisol expression, is further aggravated in the presence of job strain [42]. It has been suggested that exposure to job strain can deregulate neuroendocrine activity and, over the long term, may lead to cardiovascular diseases [42].

In short, the majority of studies indicate a positive correlation between stressors and cortisol. Concentrations of the hormone, especially in the morning, are strongly related to work stress and sleep loss [40, 43]. Therefore, it can be asserted that endocrine alterations are influenced by shift work, sleep, or circadian system changes or by a combination of these factors. The articles selected for cortisol and shift work are pointed in Table 2.

### 3.2. Obesity and Shift Work

A large portion of the global population is suffering the negative health effects of industrialization and computerization. An increase in sedentarism is evident along with a change from normal-weight to overweight and obesity. Today, obesity ranks among one of the leading causes of morbidity and mortality in industrialized countries [57, 63].

Besides the well-known risks, such as poor diet and sedentarism, shift and night work have been similarly associated with weight gain. In parallel, short sleep duration and long working hours have been observed, fuelled by economic competitiveness and globalization [62]. Thus, it has been suggested that the approach of studies on obesity must be systematic while obesity prevention should include environmental factors as its main determinants.

Epidemiologic evidence shows that shift and night work are associated with elevated risk of metabolic disorders, perhaps as a result of poor physiological adaptation to sleep loss, leading to chronic sleepiness and feeding at unfavorable circadian times [37], which in turn contribute to chronic circadian misalignment.

As mentioned earlier, shift and night work generally reduce the opportunities to practice physical activity. Moreover, the frequency of meals is also typically reduced whereas night-time snacks tend to increase. Qin et al. [53] found that over 50% of the total energy consumed by shift or night workers is consumed during the evening or night. Both the quality and quantity of foods consumed might be influenced by shift or night work and traditional meals at home discontinued. Also, nocturnal digestion is less efficient owing to circadian rhythmicity [52].

From a biological standpoint, the body is unprepared for night-time calorie intake [52]. Triacylglycerol (main lipid component of dietary fat) has shown circadian rhythmicity, with higher levels at night than during the day [52]. Night-time eating triggers a greater response of triacylglycerol compared to day-time eating. This may be the result of lower insulin response at night. Insulin is an important activator of the lipoprotein lipase, an enzyme found on capillary walls responsible for lipid hydrolysis/degradation and involved in the depuration of triacylglycerol. Therefore, a reduction in the sensitivity of insulin may result in a similar reduction in the activity of lipoprotein lipase. These results were corroborated by the study of Al-Naimi et al. [54], in which misalignment of circadian rhythms among night workers was associated with increased insulin resistance and lipid intolerance.

Appetite-regulating hormones, particularly leptin (hormone stimulating anorexigen hormones) and ghrelin (hormone acting as orexigen), have been the focus of studies aimed at better elucidating the physiopathology of obesity. These hormones, via stimulatory and inhibitory signals to the CNS, especially the hypothalamus, are essential elements in controlling appetite and feeding behavior [60].

Some studies have shown that high body mass index is an important predictor for high leptin concentrations [60]. Deficiency in the transport of leptin to the cerebral spinal fluid and/or changes in its receptors or, more rarely, genetic mutations can increase leptin levels, a condition called hyperleptinemia [58].

By contrast, high body mass index is correlated with low ghrelin levels [60]. Low ghrelin concentration can result from constant exposure to leptin, similar to the process in the hyperleptinemia condition [58]. Other explanations put forward for the low ghrelin levels observed in obese subjects include the action of leptin reducing ghrelin release, as a response to constant, high food intake, a common pattern among obese individuals, or in patients with hyperinsulinemia [56].

Another aspect related to shift work and directly associated with weight gain is the change in sleep patterns. Epidemiologic studies reveal a causal link between reduced sleep duration/shift work and metabolic disorders [61, 64]. Sleep deprivation can result in an additional daily energy intake of around 350–500 kcal and increase in the intake of calories derived from snacks [75].

Some studies have shown that individuals with short sleep duration have significantly lower sera leptin and higher sera ghrelin levels, suggesting that sleep deprivation can affect the peripheral regulators of hunger [59]. Both lack of sleep and changes in sleeping time can affect the concentrations of these hormones. This might explain the association between short sleep duration and obesity observed in studies conducted among different populations. Moreover, a study in 4,878 truck drivers showed that this combination (short sleep duration and obesity) was independently associated with several healthcare problems, including high levels of cholesterol, glucose, snoring, and hypertension [61].

Thus far, several studies have shown that changes in the sleep/wake cycle and circadian misalignment may alter concentrations of leptin and ghrelin [37, 55, 59]. With increased adiposity, there is a concomitant rise in leptin levels, where high leptin concentrations in overweight and obese individuals indicate leptin resistance [58, 60]. The study by Marqueze et al. [64] suggested that high leptin levels detected in irregular-shift workers may have been due to circadian misalignment.

In summary, aspects such as time of day, frequency, and regularity of meals as well as desynchronization of circadian rhythms can affect energy metabolism and body weight control, favoring the development of obesity.

It can be suggested that shift work constitutes an independent risk factor for weight gain and hence obesity is directly associated with circadian disruptions. These results indicate that shift and night work should be considered when attempting to prevent weight gain, with sleep representing a health pillar as important for controlling weight as the practice of physical exercise and a balanced diet.

The studies outlined underscore the need to focus on the issue of shift and night work in programs for obesity control in workers' health. Articles selected for obesity and shift work are pointed in Table 3.

### 3.3. Metabolic Syndrome, Insulin Resistance, and Diabetes and Shift Work

The literature published in recent years reveals numerous studies addressing the relationships between shift/night work and metabolic syndrome. These studies include the relationship between shift work and insulin resistance, diabetes, and dyslipidemias, as presented below.

#### 3.3.1. Insulin Resistance

There is a clear association between misalignment of circadian rhythms and insulin resistance. The mechanism underlying this resistance is not fully understood, but a combination of factors related to shift work has been implicated, such as changes in hormone levels and eating at times unfavorable for digestion [9].

Melatonin has been implicated as a key factor for the synthesis, secretion, and action of insulin. The action of melatonin also regulates the expression of transporter glucose type 4 (GLUT 4) or triggers phosphorylation of the insulin receptor. Therefore, a reduction in melatonin can be associated with an increase in insulin resistance. This may explain the reasons for some researchers to recommend melatonin supplementation for shift workers [8].

Laboratory-based experiments have shown that individuals who sleep outside normal times have reduced sensitivity to insulin, without a commensurate increase in insulin secretion [9]. The cited study supports the notion that melatonin, as well as insulin, is involved in this process [8]. The risk of a high *β*-cell activity was increased almost threefold in shift workers who work at night and very early shift [10, 65].

Eating outside the normal time of the activity period, occurring in the feeding patterns of shift workers, might lead to a circadian misalignment, although further studies are needed to give more evidence about the role of feeding as a zeitgeber [19]. This stimulation pathway may trigger the development of diseases such as insulin resistance, independently of factors such as job strain and physical activity [7, 11].

#### 3.3.2. Diabetes

Diabetes mellitus is a metabolic disorder characterized by chronic hyperglycemia due to insulin deficiency or resistance, or both. Diabetes mellitus leads to both micro- and macrovascular complications. The measure of fraction of glycosylated hemoglobin (HbA1c) (normal value <6,5%) and fasting blood sugar ≤ 126 mg/dL is often diagnostic criteria for diabetes mellitus [13].

Previous studies have suggested a twofold greater risk for developing type 2 diabetes mellitus [13] also had poor glycemic control [66] among shift workers compared to nonshift workers [66].

No clear association, however, was observed between seasonal shift work and diabetes mellitus [13]. Pooling these results and stratifying for age reveal that the association between continuous shifts and diabetes mellitus was most marked in subjects older than 45 years. This finding may indicate that the adverse health effects of shift work intensified with years of service [13].

It is likely that these adverse effects on the health of shift workers emerge when the workers have been engaged in shift work for prolonged periods (on continuous shifts) as opposed to a limited period (on seasonal shifts). In addition, for insulin-dependent diabetics, this work schedule leads to changes in meal times and consequently in timing of insulin doses, which needs to be changed without inducing episodes of hypoglycemia. This may explain the poorer glycemic control among this group. The explanation for these associations remains unclear. However, a circadian misalignment seems to occur by continuous, long-term shift work, possibly leading to the development of diseases such as type 2 diabetes mellitus, insulin resistance and weight gain, and a negative impact on sleep [68], practice of exercise [69], eating habits [70], and stress [71].

#### 3.3.3. Dyslipidemias

Shift and night work have also been associated with a higher risk of developing dyslipidemias. Karlsson et al. [72] found a relationship between metabolic disorders and shift/night work involving 1,324 individuals. A higher percentage of shift workers had elevated triglyceride levels and low HDL concentrations compared to day workers. After adjusting for age, socioeconomic factors, physical exercise, smoking, social support, and job stress, shift workers exhibited double the likelihood of low HDL concentrations (OR 2.02). High triglyceride levels were also significantly associated with both shift and night work.

Sookoian et al. [74], in a study assessing the effects of rotating shift work on biomarkers of metabolic syndrome and inflammation, found that this type of work promoted higher systolic blood pressure and greater insulin and triglyceride levels compared to day work.

La Sala et al. [73] found that risk of developing metabolic syndrome in shift workers was fourfold that of day workers. Sookoian et al. [74], however, identified a risk of 1.51, independent of age and physical activity.

In summary, according to the literature, several factors are responsible for the increased risk of developing metabolic disorders. A number of authors have cited circadian misalignment due to inversion of work hours, sleep, and meal times as a factor responsible for this association [72–74]. Articles selected for insulin resistance, diabetes, metabolic syndrome, and shift work are pointed in Table 4.

## 4. Final Remarks

In summary, recent studies on metabolic processes have increasingly revealed their complexity. The physiological changes induced in workers who invert their activity-rest cycle in order to fulfill work times include disturbances of these processes. Reduced production of melatonin due to light exposure during night shifts appears to have a major effect on energy metabolism in these workers, exerting a negative impact on their health.

Meal times and content of meal, as well as the practice of physical exercise, should suit work hours. Furthermore, strategies for avoiding stressors in the work environment and care over the quality of sleep might minimize problems resulting from shift work.

## Figures and Tables

**Table 1:** Number of articles and criteria of inclusion, according to the keywords used in this review.

Keywords	Number of articles found	Final number of articles^*^
Obesity AND shift work	235	15
Diabetes AND shift work	73	8
Metabolic syndrome AND shift work	67	3
Cortisol AND shift work	56	16
Insulin resistance AND shift work	52	4

^∗^Criteria of inclusion: Papers published between January/2003 and March/2014; studies with humans; english language papers, quality of each study (best-evidence synthesis), type of shift (rotating and/or night shift).

**Table 2:** Articles selected for Cortisol and shift work, published between 2003 and 2014.

Author (year)	Population	Main result
Dijk et al. (2012) [35]	Simulating night shift	Individual differences in amplitude of the melatonin rhythm were correlated with body temperature and cortisol.
Korompeli et al. (2009) [36]	Rotating and day shift work	The mean reduction of cortisol level between the two measurements was greater for the rotating than morning shift group.
Scheer et al. (2009) [37]	Simulating night shift	Daily cortisol rhythm was reversed. Subjects exhibit high levels of postprandial glucose responses.
Harris et al. (2010) [38]	Rotating shift (12-h day shift and 12-h night). Two weeks working followed by 4 weeks off work	Cortisol rhythm went back towards a normal rhythm in the second week, but it was not returned completely to normal levels when the workers returned home for the 4 weeks off period.
Griefahn and Robens (2010) [39]	Simulating night shift	The increased of the CAR might be the anticipation of the upcoming demands.
Ulha et al. (2011) [40]	Irregular shift and day shift workers	The concentration of cortisol declines throughout the day, reaching its lowest level during the night for day shift workers. Levels of CAR were higher on work days compared to days-off, for day shift workers. CAR levels were similar between work days and days-off for irregular shift workers.
Machi et al. (2012) [41]	Night and day shift workers	Morning cortisol peak was decreased or delayed after a night shift.
Wong et al. (2012) [42]	Rotating shift workers and day shift workers	High job strain elevated daily cortisol levels.
Nakajima et al. (2012) [43]	24-h work shift and over the subsequent day-off	Early morning cortisol levels were attenuated after work.
Diez et al. (2011) [44]	Morning and afternoon shifts	Flattening of cortisol morning-evening difference.
Williams et al. (2005) [45]	Rotating shift (early morning-shift, dayshift, and control days).	Cortisol levels on waking were lower in the early morning-shift.
Kudielka et al. (2007) [46]	Day x night shift workers	Cortisol levels in permanent night workers seemed to be blunted during night work and days-off. Circadian cortisol levels were not disturbed in former night workers who recently switched to fast rotating shift schedule.
Boquet et al. (2004) [47]	Rotating shift work: clockwise x counterclockwise direction.	No significant differences of rotating shift condition for cortisol or melatonin.
Vangelova (2008) [48]	Rotating shift	Higher salivary cortisol during morning and night shifts in the backward rotating group.
de Valck et al. (2007) [49]	Rotating shift: fast-forward versus a slow-backward rotating shift	Salivary cortisol did not significantly differ between the fast-forward and the slow-backward rotation shift systems.
Looser et al. (2010) [50]	Day shift	Associations between cortisol levels and heart rates, during periods of high stress.
van de Werken et al. (2014) [51]	Simulating Shift work	Exposure to stress, as in shift work, is predicted to result in abnormal cortisol levels.

**Table 3:** Articles selected for Obesity and shift work published between 2003 and 2014.

Autors (year)	Population	Main result
Holmbäck et al. (2003) [52]	Simulated shiftworkers	Insulin, PP, TSH, fT4, cortisol and leptin responses to meal intake differed with respect to time of day. The decreased evening/nocturnal responses of cortisol and PP to meal intake indicate that nocturnal eating and night work might have health implications.
Qin et al. (2003) [53]	Shiftworkers and dayworkers	Strong association between glucose and insulin in the diurnal lifestyle group after meals was damaged in the nocturnal lifestyle group. It was suggested that nocturnal life leads to the impairment of insulin response to glucose.
Al-Naimi et al. (2004) [54]	Simulated shiftworkers	Sequential meal ingestion has a more pronounced effect on subsequent lipid than carbohydrate tolerance.
Fogteloo et al. (2004) [55]	Simulated shiftworkers and dayworkers	The dispersion of food intake over 24 h affects the diurnal leptin rhythm. These changes could not be attributed to changes in circadian timing or energy balance.
Erdmann et al. (2005) [56]	Dayworkers	Leptin could be of importance for suppression of basal ghrelin during moderate weight gain in normoinsulinemic subjects, whereas hyperinsulinemia but not leptin is responsible in more severe obesity. Postprandial suppression of ghrelin is attenuated by as yet unknown mechanisms that are related to body weight but not to insulin or type 2 diabetes.
Flegal et al. (2005) [57]	Shiftworkers and dayworkers	Underweight and obesity, particularly higher levels of obesity, were associated with increased mortality relative to the normal weight category.
Langenberg et al. (2005) [58]	Shiftworkers and dayworkers	Ghrelin, adiponectin, and leptin do not predict weight gain beyond reflecting the influence of attained body size on future changes in weight or body mass index
Shea et al. (2005) [59]	Simulated shiftworkers and dayworkers	Alterations in the sleep/wake schedule would lead to an increased daily range in circulating leptin, with lowest leptin upon awakening, which, by influencing food intake and energy balance, could be implicated in the increased prevalence of obesity in the shift work population.
Monti et al. (2006) [60]	Shiftworkers and dayworkers	Leptin increases and ghrelin decreases were linear over the five BMI categories, suggesting there is no threshold of BMI where the hormone levels change abruptly.
Moreno et al. (2006) [61]	Shiftworkers and dayworkers	Short sleep duration as well as age >40 years are independently associated with obesity.
Chaput et al. (2007) [62]	Shiftworkers and dayworkers	Short sleep duration does not predict an increased risk of being overweight/obese in older women.
Gates et al. (2008) [63]	Shiftworkers and dayworkers	The relationship between BMI and presenteeism is characterized by a threshold effect, where extremely or moderately obese workers are significantly less productive than mildly obese workers.
Scheer et al. (2009) [37]	Simulated shiftworkers and dayworkers	Adverse cardiometabolic implications of circadian misalignment, as occurs acutely with jet lag and chronically with shift work.
Marqueze et al. (2013) [64]	Shiftworkers and dayworkers	Truck drivers are exposed to cardiovascular risk factors due to the characteristics of the job, such as high work demand, long working hours and time in this profession, regardless of shift type or leisure-time physical activity.

**Table 4:** Articles selected for insulin resistance, diabetes, metabolic syndrome and shift work, published between 2003 and 2014.

Autors (year)	Population	Main result
Esquirol et al. (2012) [10]	Shiftworkers and Dayworkers	Shiftworkers were characterized as having significantly higher triglycerides and free fatty acids and normal but lower blood glucose. The risk of a high *β*-cell activity was increased in shiftworkers. Shiftworkers had lower insulin sensitivity.
Leproult et al. (2014) [9]	Simulating Shiftworkers	Insulin sensitivity significantly decreased after sleep restriction, without a compensatory increase in insulin secretion, and inflammation increased.
Li et al. (2011) [19]	Shiftworkers	Compared with the day workers, shift workers had a significantly higher risk of metabolic syndrome (MetS).
Padilha et al. (2010) [65]	Night shift and Day shift and Early day shift Workers	The early morning group had the highest concentrations of cortisol and tended to have insulin resistence.
Esquirol et al. (2009) [11]	Rotating Shift and day Shift Workers	Shift work remained associated with metabolic syndrome, after taking into account potential covariates like job strain, physical activity, quantitative dietary parameters, and meal distribution.
Pan et al. (2011) [12]	Rotating night shifts	Long duration of rotating night shift work is associated with an increased risk of type 2 diabetes in women.
Ika et al. (2013) [13]	Day shift and continuous rotating shift work and seasonal rotating shift wok.	Compared with non-shift workers, the risk of diabetes mellitus was increased among continuous shift workers, whereas its effect is limited among seasonal shift workers.
Young et al. (2013) [66]	Day shift and night shift and rotating shift work as shift work together.	Poorer control of diabetes was associated with working shifts.
Kroenke et al. (2007) [67]	Rotating night-shift work.	Women working more hours per week had an elevated risk of diabetes.
Buxton et al. (2012) [68]	Free running and sleep restriction laboratory simulation.	Prolonged sleep restriction with concurrent circadian disruption decreased resting metabolic rate, and increased postprandial plasma glucose.
Monk and Buysse (2013) [69]	Night and rotating shift.	Shift work showed an increased proportion of self-reported diabetes with OR of about 2 when compared non-shift work.
Wirth et al. (2014) [70]	Day, night and rotating shift.	Higher pro-inflammatory diets observed among shift workers compared to day-working.
Eriksson et al. (2013) [71]	Night and rotating shift.	The risk of type 2 diabetes was increased in women who work in shift.
Karlsson et al. (2003) [72]	Shiftworkers and dayworkers	It was found an association between shift work and lipid disorders.
la Sala et al. (2007) [73]	Shiftworkers and dayworkers	Night shift work is associated with a greater risk to develop metabolic syndrome in workers healthy in baseline conditions.
Sookoian et al. (2007) [74]	Shiftworkers and dayworkers	Higher risk of developing metabolic syndrome in shift workers, independent of age and physical activity.
